# Enhancing security of incoherent optical cryptosystem by a simple position-multiplexing technique and ultra-broadband illumination

**DOI:** 10.1038/s41598-017-17916-8

**Published:** 2017-12-20

**Authors:** Sujit Kumar Sahoo, Dongliang Tang, Cuong Dang

**Affiliations:** 1 Centre for OptoElectronics and Biophotonics (OPTIMUS), School of Electrical and Electronic Engineering, The Photonics Institute (TPI), Nanyang Technological University Singapore, 50 Nanyang Avenue, Singapore, 639798 Singapore; 2 Department of Statistics and Applied Probability, National University of Singapore, Singapore, 117546 Singapore

## Abstract

A position-multiplexing technique with ultra-broadband illumination is proposed to enhance the information security of an incoherent optical cryptosystem. This simplified optical encryption system only contains one diffuser acting as the random phase mask (RPM). Incoherent light coming from a plaintext passes through this nature RPM and generates the corresponding ciphertext on a camera. The proposed system effectively reduces problems of critical alignment sensitivity and coherent noise that are found in the coherent illumination. Here, the use of ultra-broadband illumination has the advantage of reducing the speckle contrast that makes the ciphertext more complex. Reduction of the ciphertext size further increases the strength of the ciphering. Using the spatial decorrelation of the speckle pattern we have demonstrated a position multiplexed based cryptosystem, where the ciphertext is the superposition of uniquely encrypted texts from various spatial positions. These unique spatial keys are utilized to decrypt the plaintext located at different spatial positions, and a complete decrypted text can be concatenated with high fidelity. Benefiting from position-multiplexing, the information of interest is scrambled together by a truly random method in a smaller ciphertext. A high performance security for an optical cryptosystem has been achieved in a simple setup with a ground glass diffuser as a nature RPM, the broadband incoherent illumination and small position-multiplexed ciphertext.

## Introduction

As the development of the computer science and information technology, the information safety has become more challenging and drawn a lot of attention in recent years. Optical encryption technology has been commonly investigated due to the advent of parallel signal processing, multi-dimensional operations and increasing computational power^1–12^. The pioneer work of the double random phase encoding (DRPE) for optical encryption technology was proposed by Refregier and Javidi in 1995^1^. Since then many extended optical encryption algorithms and schemes, such as fractional Fourier domain^3,4^, and Fresnel domain^5^, have been reported to improve the security strength and enlarge the storage capacity. Similar to the original DRPE algorithm, these methods utilize two independent RPMs as the security key to convert the plaintext into a stationary and seemingly white noise. However, the use of coherent illumination in these conventional methods is a drawback. Not only encryption system, any optical systems based on the phase of coherent illumination are highly sensitive to the optical misalignment and unavoidable coherent artifact noise. To avoid these problems, some interesting technologies, which originally were established with coherent illumination, have been redeveloped for the incoherent illumination, such as Fresnel incoherent correlation holography^13,14^, incoherent digital holographic adaptive optics^15^, some incoherent optical correlators^16,17^.

Similarly, incoherent illumination has also been utilized for optical cryptosystems^18–20^. Use of a simple optical diffuser as an RPM was proposed to greatly reduce the complexity of the system and to decrease the errors generated from the coherent artifact noise^18,19^. Like other optical cryptosystems, the incoherent illumination based optical cryptosystems can also prone to ciphertext-only attack (COA)^21^, known-plaintext attack (KPA)^22,23^, chosen-plaintext attack (CPA)^24,25^, brute force attack^25^, and chosen-cyphertext attack (CCA)^26^, etc. Among these, COA is the hardest attack, which requires decryption of the ciphertext without any additional information. However, recent findings in the field show that diffuser based incoherent optical cryptosystems are vulnerable to COA^27^, because the ciphertext’s autocorrelation is essentially similar to the plaintext’s autocorrelation. One could recover the plaintext without knowledge of the security key by employing the phase-retrieval algorithm. The basic principle relies on the two optical phenomena: (1) completely random speckles generated by a point source through a scattering medium and (2) the memory effect of a scattering medium. The former implies the autocorrelation of the point’s speckle pattern (or point spreading function, PSF) is an impulse function^28^. The optical memory effect states that light from nearby points on the object will generate nearly identical but shifted random speckle patterns on the other side of a scattering medium (i.e. shifted PSFs). The memory effect implies the shift-invariant PSF within memory effect region of the system. Hence, the autocorrelation of the object within the memory effect region is preserved through the scattering medium. Increasing security of the incoherent illumination based optical cryptosystems is crucial.

In this paper, we propose few steps to improve the information security. Our simple optical image encryption setup contains only one diffuser to scatter light coming from various spatial objects (i.e. plaintexts) and generate a scrambled speckle pattern (i.e. ciphertext) on the camera. The first step is to use an ultra-broadband illumination. This ultra-broadband spectrum would seriously decrease the performance of the previous COA technique because the illumination bandwidth is significantly larger than the diffuser’s speckle correlation spectral bandwidth. The speckle patterns produced by multiple wavelengths in this very large bandwidth could not stay correlated^28–30^. Therefore, it would deteriorate the condition of nearly equivalent autocorrelation between the plaintext and its ciphertext, reducing the security risk from COA in the previous incoherent cryptosystems. The second step is to reduce the ciphertext size or the sensor size. An accurate estimation of the object autocorrelation from its speckle images requires larger image, because the empirical spatial decorrelation among the speckles (i.e. the impulsed autocorrelation of PSF) can only be observed with large number of speckles. Therefore a small ciphertext size will make it nearly impossible for COA to succeed unlike in the previous incoherent cryptosystems. Small ciphertext size will also reduce the storage size requirement and transmission bandwidth.

To bring an additional level of security to the ciphering and increase the encrypted information, we have used the concept of the position multiplexing^31,32^, as the third step. It goes in conjunction with the step to reduce the ciphertext size. The speckle patterns produced by the diffuser is the convolution of the object with the incoherent PSF. The method will work when the PSF is shift-invariant, i.e. the object is within the memory effect region of the scattering medium^28^. Each pixel of the output image contains the object information multiplexed in a random way. Therefore, we just need to have a small center portion of the output image as a ciphertext which is even much smaller than the full plaintext. Such a multiplexing technique and a small size speckle pattern will make it impossible to estimate the plaintext’s autocorrelation for COA. As the information of spatial plaintexts is mixed and speckles from multiple positions are overlapped, only the authorized user with correct spatial keys could decrypt corresponding pieces of information. Our proposed approach shows a higher security level and more encrypted information for incoherent optical cryptosystem.

## Principles and Simulation Results

### Incoherent optical cryptosystem

The incoherent optical cryptosystem relies on the linear shift invariant property of the diffuser, which is also know as the memory effect^33^. The image of an incoherent object within this memory effect can be expressed as its convolution with a point spreading function (PSF) as follows.

1 $$I(x,y)=O(x,y)\ast PSF(x,y)$$

where the intensity image *I* or ciphertext is a speckle pattern as the output, *O* is a object intensity or plaintext as the input, the *PSF* plays the role of security key, * is the convolution operation, and *x* or *y* corresponds to the coordinate along *x* or *y* direction at the output plane. Equation (1) is the main essence of the incoherent cryptosystem^18,19^, which could be approximated with discrete convolution in the pixel space as follows.

2 $$I(x,y)=\sum _{i,j}O(i,j)PSF(x-i,y-j)$$

For the PSF (i.e. the security key) and the plaintext, we can calculate one of them if we know the other through the deconvolution process as:

3 $$O=F{T}^{-1}(\frac{FT(I)FT{(PSF)}^{c}}{\parallel FT(PSF){\parallel }^{2}})$$

or

4 $$PSF=F{T}^{-1}(\frac{FT(I)FT{(O)}^{c}}{\parallel FT(O){\parallel }^{2}})$$

where (.)^*c*^ is the complex conjugate, *FT* and *FT* ^−1^ are the Fourier transform operation and the inverse Fourier transform operation respectively. Equation (3) presents the decryption process, where the decrypted text is derived with the security key (PSF).

Many popular attacks such as KPA, CPA, CCA… mainly rely on estimating the key (i.e. PSF) with the knowledge of the plaintext as described in equation (4). However, such attacks is unlikely because of the requirement to have access to the system to known the plaintext. Our position multiplexing technique will be discussed in the next subsection to protect the encrypted text from these attacks. Recently, COA has been demonstrated for diffuser based incoherent optical cryptosystem^27^, because the ciphertext’sautocorrelation is essentially similar to the plaintext’s autocorrelation. It can mathematically expressed as follows.

5 $$\begin{array}{ccc}[I\,\star \,I](x,y) & = & [(O\ast PSF)\,\star \,(O\ast PSF)](x,y)\\  & = & [(O\,\star \,O)\ast (PSF\,\star \,PSF)](x,y)\approx [O\,\star \,O](x,y)\end{array}$$

where $\star$ is the correlation operator. One could recover the plaintext without knowledge of the security key (PSF) by employing the phase-retrieval algorithm. However, the equation (5) completely relies on the following relationship which is based on the randomness nature of PSF.

6 $$[PSF\,\star \,PSF](x,y)\approx \delta (x,y)=\{\begin{array}{cc}1 & if(x,y)=(0,0)\\ 0 & otherwise\end{array}$$

If we can make necessary modification to the existing cryptosystem to empirically break the idealistic equation (6) we will enhance the security significantly. Two of such modifications are presented in this work: the reduction in contrast of the PSF, and reduction in the sensor size. These two factors largely impact the estimation of the speckle autocorrelation^28^.

**Figure 1:**
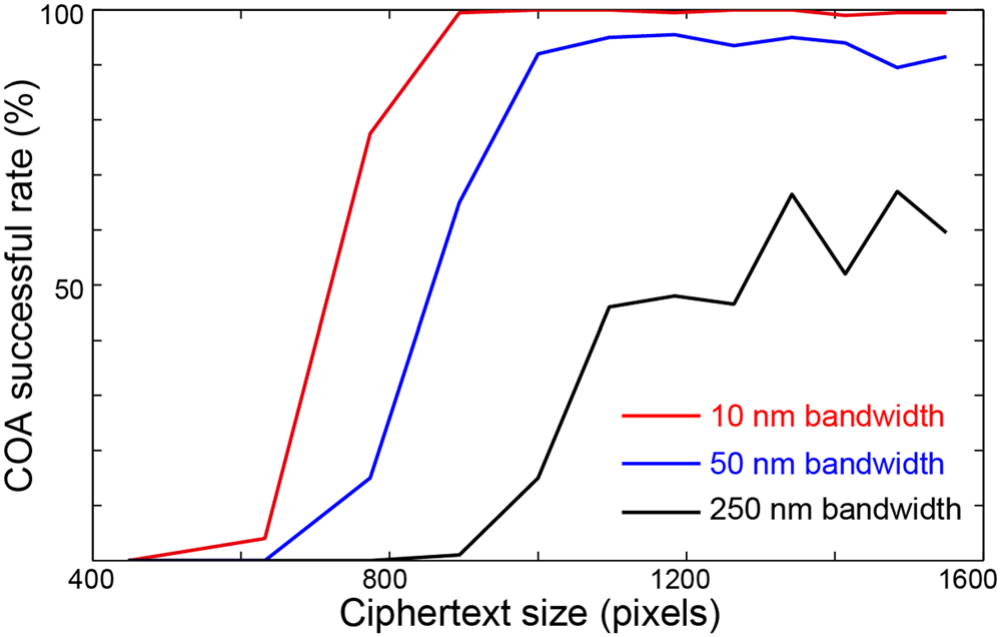
The strength against COA of incoherent optical cryptosystem with broadband illumination and small ciphertext size. Simulating the successful rate of COA as a function of ciphertext size for three different spectral bandwidths: 10 nm, 50 nm and 250 nm. Here, the number of pixels refers to one dimension of the square ciphertext.

In order to demonstrate the security enhancement agains COA, we simulate the attack for illumination at the bandwidth of 10 nm, 50 nm and 250 nm and for various ciphertext size. In our simulations, each unique random speckle pattern is generated from Rayleigh distribution^34^. Assuming the diffuser’s speckle correlation spectral bandwidth is 2 nm, we will have 1 random speckle for each 2 nm bandwidth. For the 10 nm, 50 nm and 250 nm broadband illumination, the PSFs are generated by superposition of these 5, 25 and 125 independent random speckle patterns respectively. The ciphertext is created by convolving the PSF with the desired plaintext. Then it is added with random Gaussian noise to make a signal to noise ratio (SNR) of 30 dB, and finally it is sub-quantized to 8 bit per pixel. The original ciphertext size of 1600 × 1600 pixels is then cropped at the center to generate different ciphertext size. Then, we simulate the COA by running phase retrieval algorithm mentioned in ref.^27^, which implements hybrid input-output (HIO) and error reduction (ER) method. Figure 1 shows the simulation results of COA successful rate as a function of ciphertext size for different illumination bandwidths. Increase the spectral bandwidth from 10 nm to 250 nm reduces the successful rate to 50% even at the highest ciphertext size. Decreasing the ciphertext size will reduce the successful rate and finally make the COA impossible with the ciphertext less than 400 × 400 pixels even with narrowest spectral bandwidth of 10 nm.

### Position multiplexing

**Figure 2:**
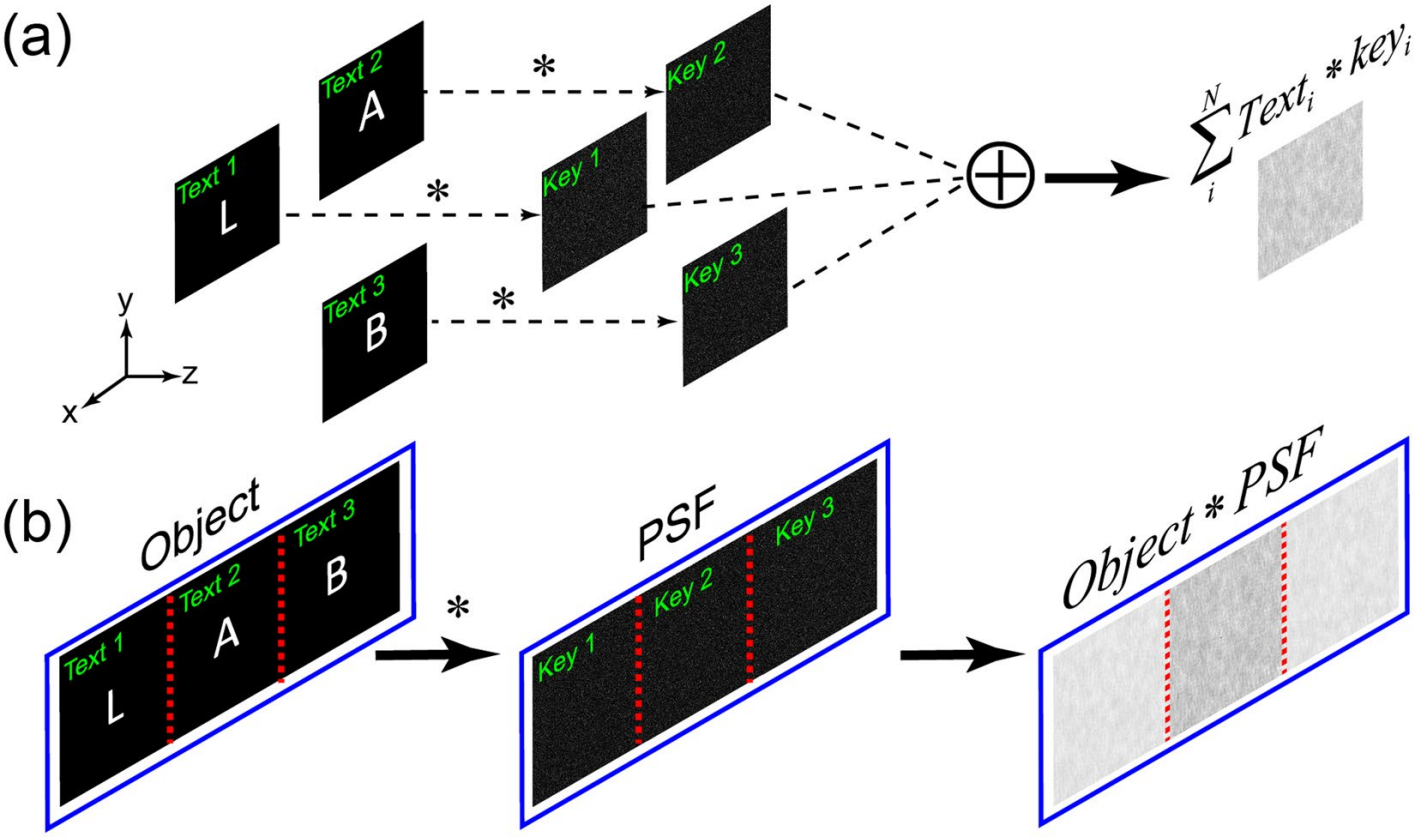
Concept of position-multiplexing technique in linear optical cryptosystems. (**a**) Each plaintext is encrypted by independent security key, then the final ciphertext is the superposition of all the encryption. (**b**) Artificially create the multiple small PSFs (the security keys) by cropping multiple parts from a full-scale PSF of a single optical diffuser to simplify the approach. This also reduces the ciphertext size to the size of the small PSFs.

We introduce an additional level of security to the incoherent optical cryptosystem with a practical position multiplexing technique. The concepts of position and wavelength multiplexing have been proven effective in coherent optical cryptosystems^31,32^. The motivation is to embed more number of uniquely separable ciphertexts into a single ciphertexts.

7 $$I(x,y)=\sum _{k}{O}_{k}(x,y)\ast PS{F}_{k}(x,y)$$

where *PSF*_*k*_ is the corresponding encryption key for the text object *O*_*k*_. Figure 2a illustrates the principle of position multiplexing concept in incoherent optical cryptosystem. In the deciphering process, we just need to deconvolve the multiplexed ciphertext *I* with the respective key *PSF*_*k*_ to obtain the underlying object as follows.

8 $${O}_{k}=F{T}^{-1}(\frac{FT(I)FT{(PS{F}_{k})}^{c}}{\parallel FT(PS{F}_{k}){\parallel }^{2}})$$

The aforementioned deciphering technique without cross-talk (or interference) is possible with the condition that these keys are unique and uncorrelated to each other. The multiplexing scheme can asymptotically be considered as an ideal case of orthogonal multiplexing.

9 $$PS{F}_{k}\,\star \,PS{F}_{l}=\{\begin{array}{cc}0 & {\rm{i}}{\rm{f}}\,k\ne l\\ \delta  & {\rm{i}}{\rm{f}}\,k=l\end{array}$$

where *δ* is the spatial impulse function. A details description a similar multiplexing phenomenon can be found in the recent multispectral imaging^35^, which can be considered as an instance of wavelength multipexing.

This would make it impossible for the attacker to decipher the system with or without any partial knowledge of the system. Now the autocorrelation of the ciphertext is the superposition of the autocorrelation of the individual objects.

10 $$\begin{array}{ccc}[I\,\star \,I](x,y) & = & \sum _{k}[({O}_{k}\,\star \,{O}_{k})\ast (PS{F}_{k}\,\star \,PS{F}_{k})](x,y)+\sum _{l\ne k}[({O}_{l}\,\star \,{O}_{k})\\  &  & \ast (PS{F}_{l}\,\star \,PS{F}_{k})](x,y)\approx \sum _{k}[{O}_{k}\,\star \,{O}_{k}](x,y)\end{array}$$

It is not possible to segregate the autocorrelation of the individual objects and perform the COA using phase retrieval. Many other popular attacks try to estimate the key (i.e. PSF) with the knowledge of the plaintext (equation 4). However, with position multiplexing technique, it would not be possible to directly estimate the key or the PSF for any known text *O*_*k*_, because:

11 $$PS{F}_{k}\ne F{T}^{-1}(\frac{FT(I)FT{({O}_{k})}^{c}}{\parallel FT({O}_{k}){\parallel }^{2}})=PS{F}_{k}+F{T}^{-1}(\sum _{l\ne k}\frac{FT(PS{F}_{l})FT({O}_{l})FT{({O}_{k})}^{c}}{\parallel FT({O}_{k}){\parallel }^{2}})$$

In principle, the position multiplexing would certainly add an essential security layer to the incoherent optical cryptosystem. However, the implementation of position multipexing needs complicated optical arrangements for multiple independent PSFs (Fig. 2a). A holographic recorder was proposed for multiplexing technique in coherent cryptosystem^31^. Here, we have developed a simple approach to realize the position multiplexing technique by utilizing the linear shift-invariant property of the diffuser. A schematic of our position-multiplexing realization is presented in Fig. 2b.

From of a full-scale image of the point’s speckle pattern (i.e. a full-scale PSF), we can extract multiple non-overlapping portions which plays the role of multiple independent PSFs in our multiplexing technique. The spatial decorrelation of the PSF enables the position-multiplexing of the objects which are spaced apart as the dimension of the keys (Fig. 2b). In this technique, the ciphertext with the size of PSFs is also a cropped portion of the full-scale ciphertext. The position multiplexing approach automatically reduces the ciphertext size, which enhances the security. Then we use the spatially non-overlapping windows of the full scale PSF as the security keys to extract the information of the objects at the corresponding spatial positions. The decryption process is done digitally. Each PSF in our approach is considered as a security key, generated by an unique RPM in conventional optical cryptosystem. Our position-multiplexing technique can be considered as the simplest technique to superpose the cyphertexts of different nature RPM based encryption system. In practice, the sender and the receiver have already exchanged the keys, and by using these keys, many ciphertexts can be transferred and decrypted. The receiver doesn’t need to own the RPM or any optical setup.

## Experiments and Results

**Figure 3:**
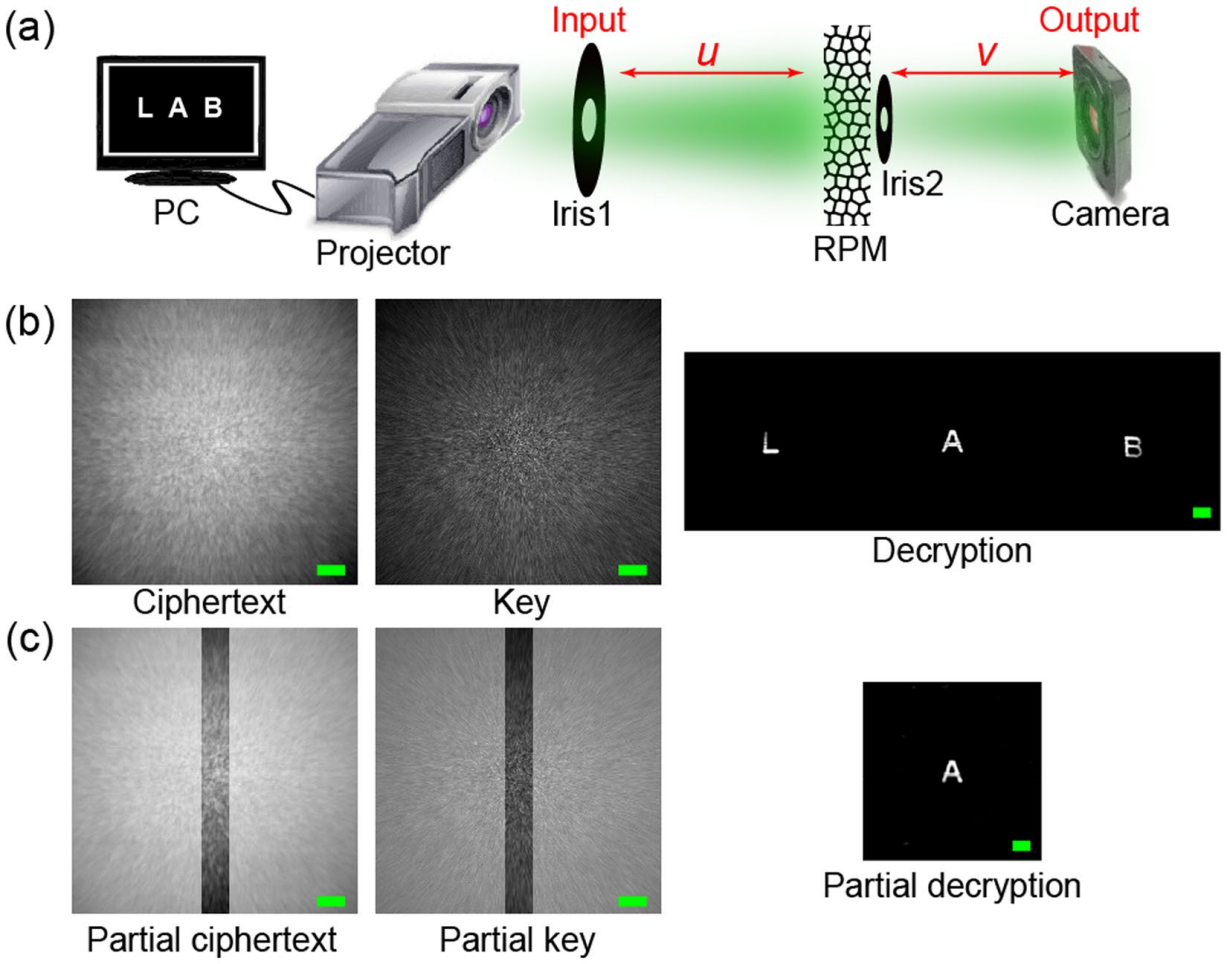
Experimental demonstration of the position-multiplexing principle in a cryptosystem with one RPM. (**a**) A plaintext with ‘LAB’ letters at different spatial positions are encrypted through one RPM and a camera records the mixed/scattered ciphertext. (**b**) Ciphertext, key and decryption as utilizing full camera image for decryption processing. (**c**) Partial ciphertext, key and decryption as using the reduced images. Scale bars: 200 pixels in ciphertexts and keys, and 20 pixels in decryptions.

The complete encryption setup is schematically shown in Fig. 3a. The point source and desired plaintext are displayed by the projector and projected at the input plane, where the iris 1 is used for minimizing the background light from the projector. We use full white-light, from 400 nm to 720 nm wavelength of the projector in our experiments. One diffuser (Edmund, 120 Grit Ground Glass Diffuser) is placed at a distance from the input plane and scrambles the original light field of the plaintext. A scientific camera (Andor Neo 5.5, 2560 × 2160, pixel size 6.5 um) is used to capture the encrypted image at the output plane. Iris 2 with about 2 mm diameter is used for obtaining an appropriate speckle intensity, grain size and signal-to-noise contrast according to the reported reference about the scattering media^28,35^. The distance from the iris 1 to RPM and distance from RPM to the camera are *u* = 210 mm and *v* = 87.5 mm, respectively. The PSF (or security key) is actually the speckle pattern generated by a point source (1 pixel in projector) in this setup.

In this work, we utilize the standard Wiener deconvolution algorithm to do decryption processing. A reconstruction with higher fidelity could be obtained by using a larger image size, as the reconstruction artefact increases with higher correlation between the noise and actual signal. This noise-signal correlation decreases as the total pixels or the image dimension increases^35^. Therefore, there is a trade-off between the image quality and the ciphering strength. For clarity, we remove the background which is set at 20% of the maximum intensity in the decrypted text. In Fig. 3b, a decrypted result is shown by taking the intensity *I* with 2048 × 2048 pixels as the ciphertext, and the PSF with the same size as the key. All the features of the large plaintext “LAB” are successfully reconstructed. In Fig. 3c, the decrypted result is shown by taking the intensity *I* with only 200 × 2048 central pixels as the ciphertext and the same area on PSF as the key. It shows that by cropping the intensity image and PSF, the peripheral objects are completely lost. Even though the partial intensity image *I* contains every information of the input object, the partial PSF could only be able to reconstruct the object specific to its position and dimension.

**Figure 4:**
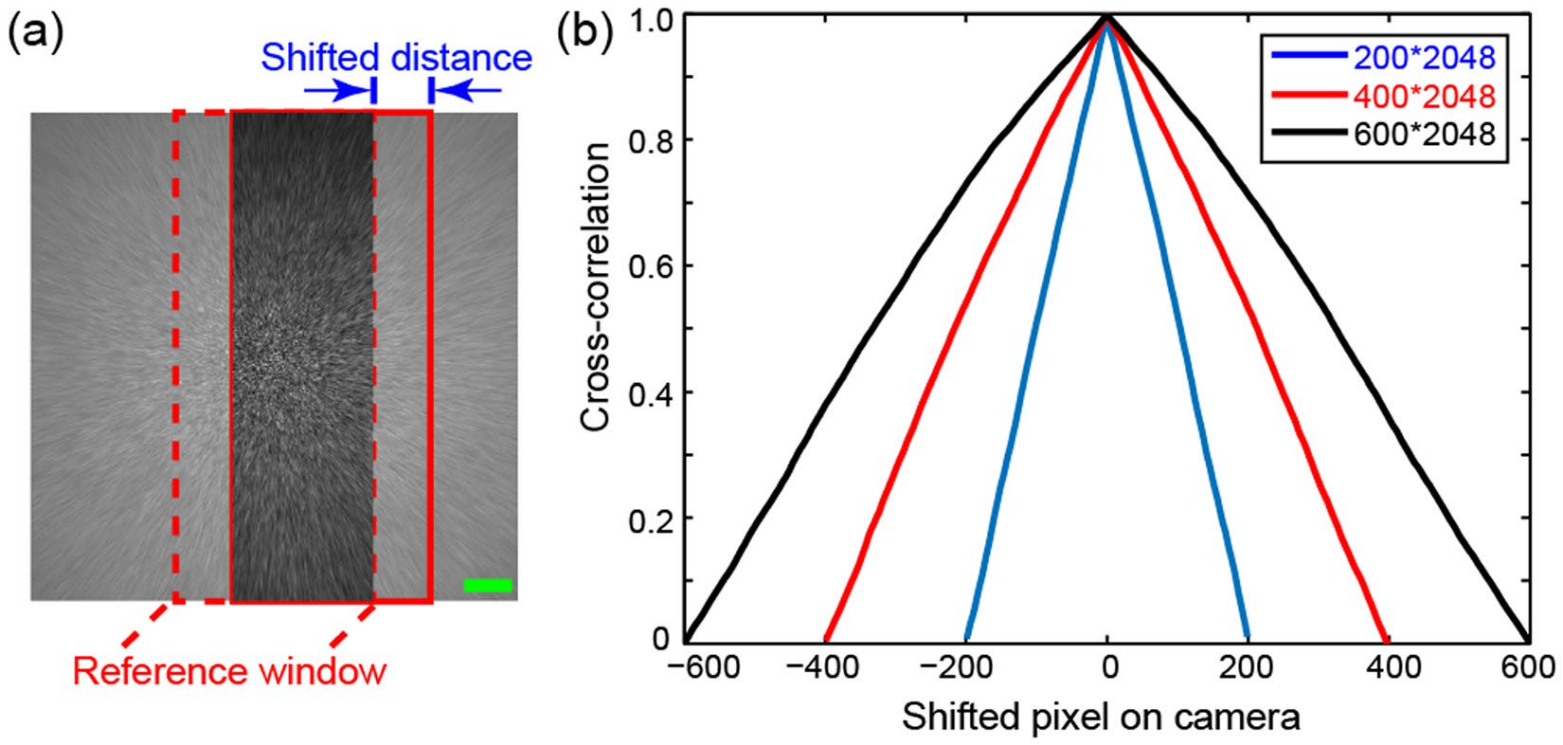
Cross-correlation between the reference window and the windows extracted at different shifts. (**a**) The measured speckle pattern of the PSF. The transparent region illustrates the correlated speckles between reference window and window extracted at a shifted position (the solid lined rectangle). (**b**) Cross-correlation coefficient between the extracted windows at different shifts for various window sizes: 200 × 2048, 400 × 2048 and 600 × 2048 pixels. Scale bar: 200 pixels.

**Figure 5:**
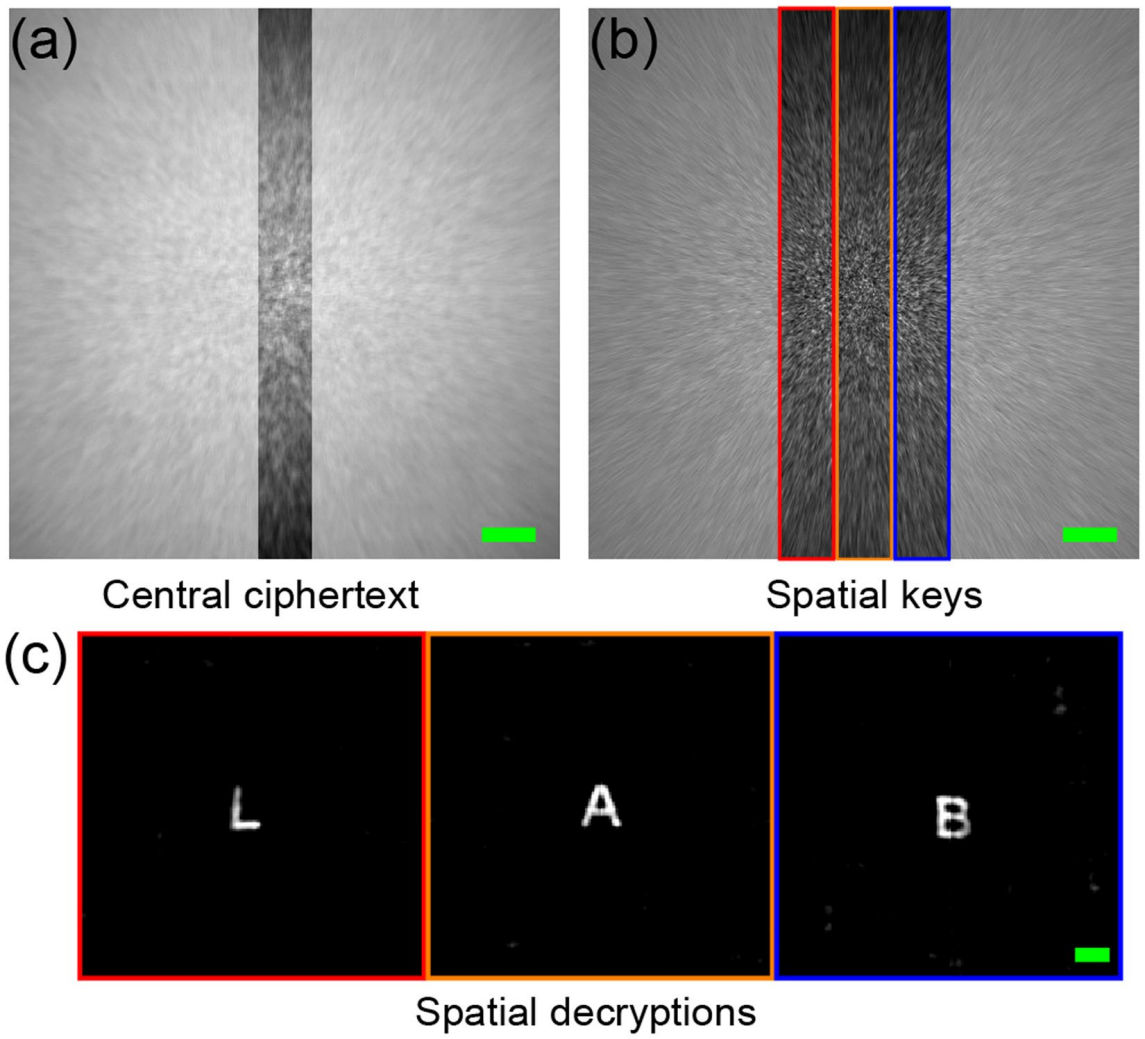
Stitched 1D decryption with various spatial keys. (**a**) Ciphertext with 200 × 2048 pixels in central part. (**b**) Various spatial keys with the same size in (**a**). (**c**) A full 1D recovery is stitched together with different spatial decryptions. Scale bars: 200 pixels in (**a**) and (**b**), and 20 pixels in (**c**).

Non-overlapped areas on the PSF generated by a diffuser are uncorrelated to each other. As a result, the reconstruction with partial PSF in Fig. 3c has no impression of the peripheral texts. In order to demonstrate this spatial decorrelation property of the PSF, we have plotted the correlation curve taking various window sizes. We first extract a window (dash-lined rectangle) from the center of the PSF and use it as the reference to compute the correlation with the windows at various horizontal shift positions (the solid rectangle) as shown in Fig. 4a. The relationship between correlation coefficient and pixel shift is plotted in Fig. 4b. The vertical size of window is fixed at 2048 pixels while the horizontal sizes of the windows are: 200, 400 and 600 pixels. The result shows a decrease in correlation of the PSF window with the shift increase then a complete decorrelation when there is no spatial overlaps (i.e. the shift is beyond the window size). Figure 3c has also demonstrated this decorrelation, where the letter “A” is clearly reconstructed without any cross-talk (or interference) from “L” and “B”. It is because the letters are more pixels apart than the width of the key and there is no cross-talk between the keys. We demonstrate the position-demultiplexing by extracting 3 non-overlapping keys with size 200 × 2048 pixels from the full-scale PSF, which is shown in Fig. 5(b). Here, the ciphertext is still the intensity *I* with central 200 × 2048 pixels, as presented in Fig. 5(a), which has the text information of all the spatial positions in the input plane. Three images are reconstructed from the single ciphertext using 3 different keys, and placed at the respective spatial position of the keys. This concatenated image is shown in Fig. 5(c) with a successful recovery of the 3 letters, similar to using full-scale ciphertext and full-scale PSF as in Fig. 3(b).

**Figure 6:**
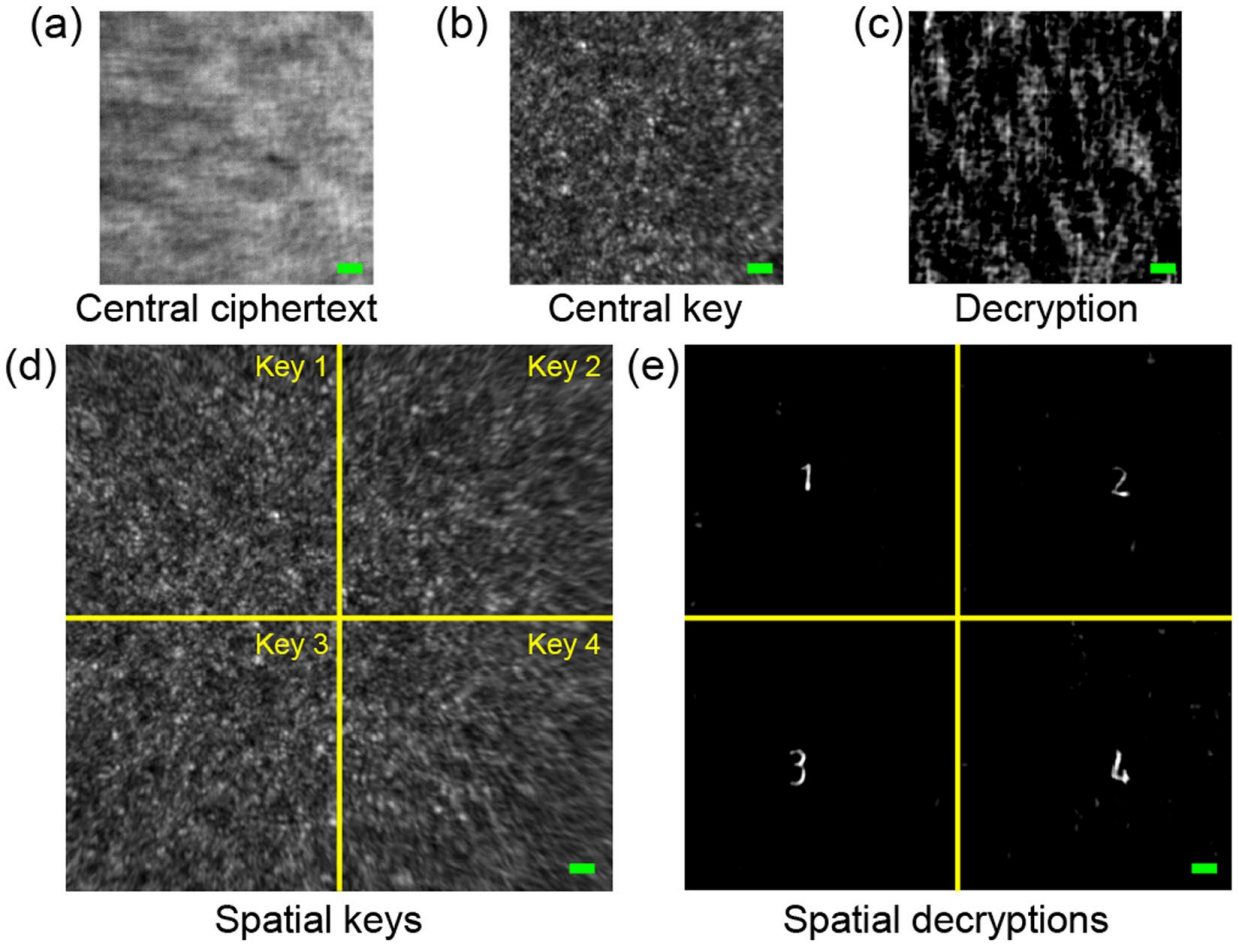
Stitched 2D decryption with various spatial keys. (**a**–**c**) Ciphertext, key and decryption with central 220 × 220 pixels. (**d**) Various spatial keys with the same size in (**a**). (**e**) A full 2D recovery is stitched together with different spatial decryptions. Scale bars: 20 pixels.

We select the centre portion of the intensity image as a ciphertext, because it has the highest signal-to-noise ratio due to the hallow effect of the speckle pattern. However, in principle, each pixel in intensity image carries all the object information as a result of convolution and the randomness of light passing through scattering media. We don’t necessarily need to have the keys aligned with the centre intensity image. In the next demonstration, we take a text object having four numbers at four corners. The central area (220 × 220 pixels) of the intensity image is taken as the ciphertext, which is shown in Fig. 6(a). Then we have taken the same central 220 × 220 pixels of the PSF as the key, which is shown in Fig. 6(b). The reconstructed image, Fig. 6(c), shows that central key decrypts no information as the central portion of the object is blank. Next, we generated four keys as spatial PSFs by dividing the central 440 × 440 pixels of the PSF, as illustrated in Fig. 6(d). The stitched decryption is displayed in Fig. 6(e), which presents a very good reconstruction of the four letters at the four corners, each with the corresponding key. In this demonstration, the background with the normalized intensity smaller than 0.4 are removed as the reduced sizes for the ciphertext and keys are much smaller than that in Fig. 3 and generate more reconstruction background noises.

To confirm the strength of our ciphering method, we run phase retrieval algorithm mentioned in ref.^27^, which implements hybrid input-output, and error reduction strategy. While the phase retrieval algorithm shows 30% successful rate with full-scale ciphertext in Fig. 3b. With the small ciphertext in Figs 3(c), 5(a) and 6(a) as inputs, the phase retrieval algorithm cannot produce any information about the plaintexts. We also attempt to derive the keys from ciphertext and plaintexts according to equation (4). For full-scale ciphertext and the plaintext in Fig. 3b, the key is derived successfully. While the small ciphertext with position multiplexing as in Figs 3(c), 5(a) and 6(a) does not allow us to derive the keys if we know some plaintexts.

## Discussion and Conclusion

RPM based optical cryptosystems falls under the category of linear systems. Linear cryptosystems are prone to various forms of attacks including COA. Certainly, nonlinear system is better for information security but it is more complex. Therefore, in this work we significantly enhanced the security of linear optical cryptosystem by a simple approach. The COA is mainly attributed to the high contrast of the ciphertext (or the speckle images), as it relies on the accurate estimation of the plaintext’s autocorrelation. Our proposed method would increase the security by reducing the speckle contrast by utilizing ultra-broadband illumination. In addition, our realization of position-multiplexing has significantly reduced the ciphertext size and made the attack more challenging. In contrast with other linear optical encryption methods, our position-multiplexing technique allows us to have a single cyphertext, which carries multiple plaintexts with different keys. This will make it significantly difficult for attackers to derive the keys with the knowledge of some plaintexts and cyphertexts as in some other attacks, such as known-plaintext attack (KPA), chosen-plaintext attack (CPA), chosen-cyphertext attack (CCA) and brute force attack. The information of multiplexing positions for these keys which determine the orders of multiple decryption texts, will add another level of security to our system. The more number of multiplexing positions will enhance the security of the system. At the same time, it will also reduce the number of pixels for the keys and cyphertext, resulting in poor recovery via deconvolution (Supplementary information). This sets the tradeoff between the reconstruction quality and the security. The gray-scale image as the plaintext is another consideration in our approach. However, decreasing the size of ciphertext and key makes the background noise higher as shown in Figs 3c, 5 and 6. We need to remove background corresponding to 20% and 40% of peak intensity in the decrypted text when the cipher text size reduces from 200 × 2048 to 220 × 220 pixels. Note that we do not need to remove background when using the full-scale ciphertext and key (2048 × 2048 pixels). In addition, the optical memory effect also reducing with the distance^28,35^, which makes the peripheral texts dimer automatically. This implies the challenge to encrypt/decrypt gray-scale images and there is a trade-off between the number of gray-scale levels with the size of ciphertext and keys (Supplementary information).

In conclusions, we demonstrate a position-multiplexing based cryptosystem to enhance the information security. This method utilizes the two fundamental properties of a scattering medium: memory effect and PSF’s spatial decorrelation. Ultra-broadband incoherent illumination and position multiplexing with small size of ciphertext image significantly enhance the security. The unique spatially distributed keys are extracted from the same PSF for decryption. Multiple plaintexts can be multiplexed by a truly random technique in a single ciphertext and sent to all users who will individually decrypted the specific texts with their corresponding keys. As the spatial information of interest are scrambled together or hidden inside the ciphertext, one can decrypt the content with multiple spatial keys but still need to know the keys’ order to arrange the multiple pieces of information.

### Data availability

The datasets generated during and/or analysed during the current study are available from the corresponding author on reasonable request.

## Electronic supplementary material

Supplementary Information
