# Supplementary material for: Enhancing security of incoherent optical cryptosystem by a simple position-multiplexing technique and ultra-broadband illumination
Source: Sci Rep. 2017 Dec 20;7:17895. doi: 10.1038/s41598-017-17916-8 (PMC5738381; doi:10.1038/s41598-017-17916-8)
Supplement: Supplementary file 1 — Supplementary Information [file 41598_2017_17916_MOESM1_ESM.pdf]

Supplementary information:

Enhancing security of incoherent optical cryptosystem by a simple position-multiplexing technique and ultra-broadband illumination

Sujit Kumar Sahoo[1,2,*,+], Dongliang Tang[1,+], and Cuong Dang[1,*]

Affiliations:

[1]Centre for OptoElectronics and Biophotonics (OPTIMUS), School of Electrical and Electronic Engineering, The Photonics Institute (TPI), Nanyang Technological University Singapore, 50 Nanyang Avenue, Singapore, 639798 Singapore

[2]Department of Statistics and Applied Probability, National University of Singapore, Singapore, 117546, Singapore.

[*] sujit@pmail.ntu.edu.sg, hcdang@ntu.edu.sg

[+]These authors contributed equally to this work.

Experiment sections for grayscale and multiplexing capability

To further demonstrate the multiplexing ability for the grayscale image, a plaintext as presented in Figure S1(a) is displayed through the projector in the same setup. There are two multiplexed positions corresponding to the position of 'AB' and 'CD' in this experiment. The letter 'B' with different grayscale ratios (with 0.3, 0.4, 0.5, 0.6, 0.8 and 1.0 of the intensity in letter 'A') are chosen to demonstrate the grayscale multiplexing and duplexing. The reconstructions with partial camera pixels and the corresponding 1D lines through the central horizontal directions are shown in figure. (b) - (g). From the figures, a dim reconstruction of letter 'B' somewhat appears when the grayscale ratio is 0.4, and a better visual 'B' could be certainly distinguished as the grayscale ratio increases. Here, we should mention that for grayscale ratio with 0.8 and 1.0, even though reconstructions with high quality could be achieved, the intensity for 'A' and 'B' might not be fully recovered. This problem comes from the fact that in real optical setup, due to the random structure of the diffuser, alignment of projector and optics, the responses with respect to the various spatial positions on the object plane might be different on the camera, especially for the intensity. In addition, the optical memory effect is also decrease with the distance from the center. Therefore, the plaintext near the center maybe have a stronger transmission and intensity on the camera and creating the challenging for gray-scale plaintext. Nevertheless, this real experiment still demonstrates the ability of grayscale multiplexing.

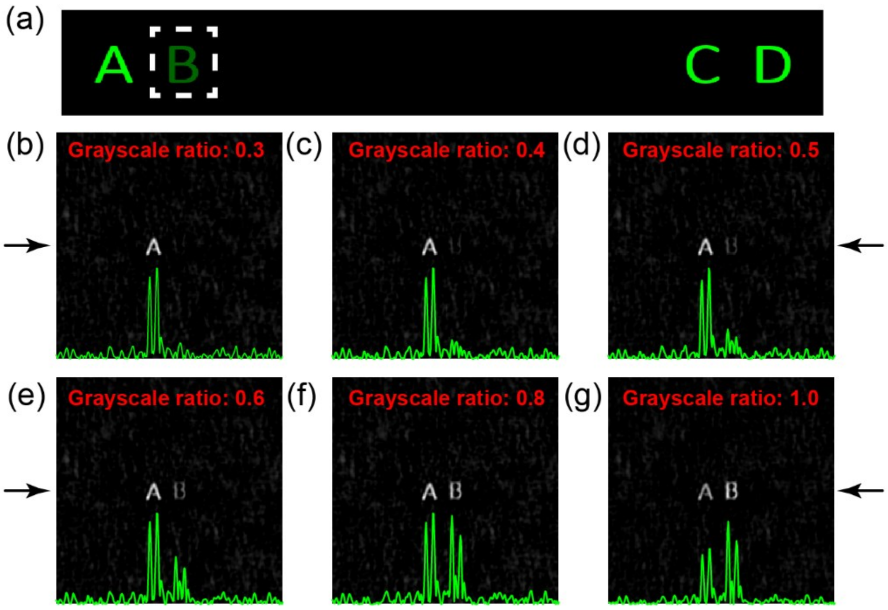

Figure S1. Experimental demonstration of the position-multiplexing method with grayscale plaintexts. (a) A plaintext with 'ABCD' letters at different spatial positions and the letter 'B' has different intensity (i.e. grayscale values: 0.3, 0.4, 0.5, 0.6, 0.8, 1.0) comparing to the intensity in other letters. (b) - (g) Decryptions with same partial camera images (300×300 displayed pixels) for various grayscale values in 'B' letter, and the corresponding 1D intensity lines along the central horizontal directions.

Next, the multiplexing capability for the number of the total encrypted plaintexts is also investigated. From the above experiments with partial camera pixels, the signal of interest could be recovered but there are some background noise surrounding the region. Those reconstructed noises mainly come from the insufficient speckle information from the plaintexts as the artificial cropping camera sizes decrease, the wavelength multiplexing from the used broadband illumination and the position multiplexing from the nearby plaintexts. Nevertheless, it is worth to check the number of the encrypted images can be multiplexed with our current configuration. In the experiments, the letter 'A' at the corner is displayed by the projector. Then a successful reconstruction with full camera image is obtained, as presented in Fig. 2(a). Next, more letters are added one by one at the right side with same distance; and the corresponding reconstructions with full-scale camera images are presented in Fig. 2(b)-(d). Due to the measured PSF corresponding to the one point in the middle of 'B' and 'C', the intensity at the central region are stronger than the surrounding region because the memory effect (i.e. correlation coefficient) is decaying with the increase of distance. Then, the reconstructions with partial camera pixels are presented in Fig2. (e)-(h). We can see that if only one plaintext is displayed by the projector, the recovered image is visual with acceptably low noise in Fig 2(e). However, after adding more plaintext of the same intensity, the corner plaintext reconstruction is poorer but still visible. The reason is that the new plaintexts near the center where the PSF is measured have higher correlation coefficient with PSF, and therefore are reconstructed with higher intensity. After adding the fourth plaintext with letter 'D', the reconstruction for this plaintext is not clear. Here we present only the multiple plaintexts in horizontal direction. If one extend the plaintexts to 2D plane as our demonstrations in Fig. 6,

more number of plaintexts can be achieved. The main limitation for the small number of the encrypted images is from the memory effect region defined by the diffuser and the capability of the camera (pixel resolution, photon capacity and bit-depth).

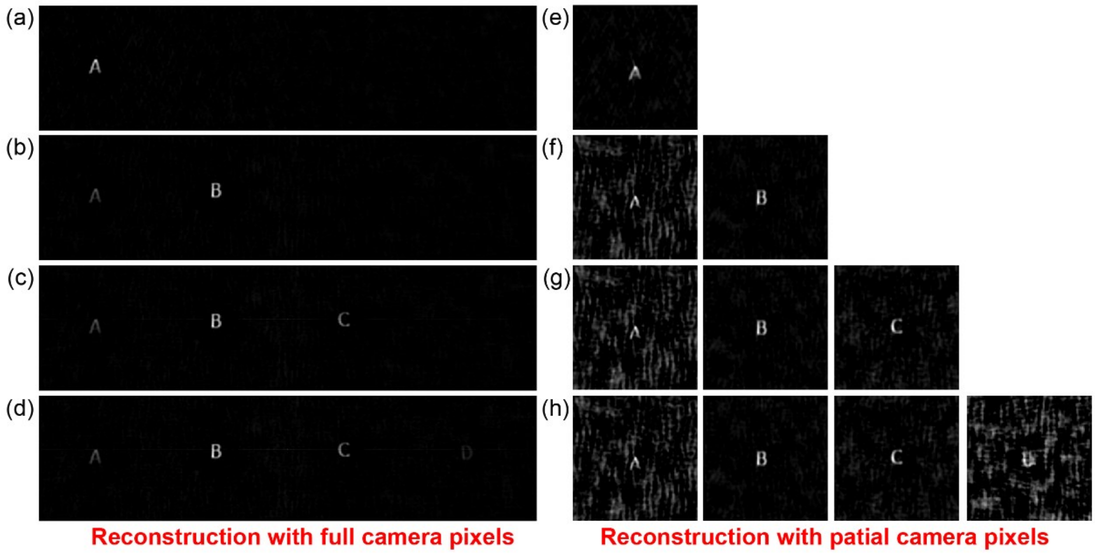

Figure S2. Experimental demonstration of the multiplexing capability for the number of encrypted plaintexts. (a) – (d) Reconstructions with the full-scale camera image (200×800 displayed pixel) when adding the plaintext from the left to right one by one with same distance. (e) - (h) Decryptions with small camera images (200×200 displayed pixels) for various spatial plaintexts, corresponding to the full-scale camera images reconstruction (a-d).
